# Comparison between Different Methods for Biomechanical Assessment of *Ex Vivo* Fracture Callus Stiffness in Small Animal Bone Healing Studies

**DOI:** 10.1371/journal.pone.0119603

**Published:** 2015-03-17

**Authors:** Malte Steiner, David Volkheimer, Nicholaus Meyers, Tim Wehner, Hans-Joachim Wilke, Lutz Claes, Anita Ignatius

**Affiliations:** Institute of Orthopedic Research and Biomechanics, Center of Musculoskeletal Research Ulm, University Hospital Ulm, Ulm, Germany; University of Notre Dame, UNITED STATES

## Abstract

For *ex vivo* measurements of fracture callus stiffness in small animals, different test methods, such as torsion or bending tests, are established. Each method provides advantages and disadvantages, and it is still debated which of those is most sensitive to experimental conditions (*i*.*e*. specimen alignment, directional dependency, asymmetric behavior). The aim of this study was to experimentally compare six different testing methods regarding their robustness against experimental errors. Therefore, standardized specimens were created by selective laser sintering (SLS), mimicking size, directional behavior, and embedding variations of respective rat long bone specimens. For the latter, five different geometries were created which show shifted or tilted specimen alignments. The mechanical tests included three-point bending, four-point bending, cantilever bending, axial compression, constrained torsion, and unconstrained torsion. All three different bending tests showed the same principal behavior. They were highly dependent on the rotational direction of the maximum fracture callus expansion relative to the loading direction (creating experimental errors of more than 60%), however small angular deviations (<15°) were negligible. Differences in the experimental results between the bending tests originate in their respective location of maximal bending moment induction. Compared to four-point bending, three-point bending is easier to apply on small rat and mouse bones under realistic testing conditions and yields robust measurements, provided low variation of the callus shape among the tested specimens. Axial compressive testing was highly sensitive to embedding variations, and therefore cannot be recommended. Although it is experimentally difficult to realize, unconstrained torsion testing was found to be the most robust method, since it was independent of both rotational alignment and embedding uncertainties. Constrained torsional testing showed small errors (up to 16.8%, compared to corresponding alignment under unconstrained torsion) due to a parallel offset between the specimens’ axis of gravity and the torsional axis of rotation.

## Introduction

Small animals such as rats and mice are becoming increasingly popular models for investigating fracture healing. Usually, *ex vivo* analyses are performed to measure and quantify the regained stiffness of the fracture callus to evaluate healing success or detect differences between different treatment groups. The quality of the newly formed bone is commonly determined by either measuring the callus strength with load-at-failure methods or by evaluating the callus stiffness with non-destructive bending or torsion test set-ups [1,2,3,4,5]. Non-destructive torsional testing is performed in rat [6,7,8,9,10] as well as in mouse experiments [11,12,13,14]. Bending is also performed in experiments for both species: three-point bending in rats [15,16,17,18] and mice [19,20,21,22,23] as well as four-point bending in rats [24] and mice [25].

Due to the small size of rat and mouse bones however, mechanical testing is often difficult and measurement errors result from incorrect alignment of the specimens in the testing apparatus [26,27]. This often leads to large standard deviations in the experimental groups and makes statistical analyses difficult [3,28]. In addition, the fracture callus sometimes shows an asymmetric arrangement, even within one experimental group, which might affect the measured results depending on the measurement protocol used [11]. This has led to a significant debate in the research community as to which type of mechanical test should be used for biomechanical characterization.

Three-point bending provides the distribution of internal loading most similar to physiology for mechanical characterization, especially for the most critical location to be investigated. However, drawbacks include its high directional dependency [29,30] and potential influence of the indentation from the test stamp on the results, since it compresses tissue mostly in the critical region of the investigated specimen (*i*.*e*. the callus site in a fractured long bone).

Torsional testing has the great advantage that the torsional stiffness it is not affected by the orientation of asymmetric calluses, and is therefore direction independent and provides a characterization of the investigated specimen as a whole [3]. The easiest and most common way to perform *ex vivo* torsion testing is to fix the long bone at the distal end in all six degrees of freedom (DOF), fix the proximal end in all DOF except torsion, apply a torsional moment to the proximal end and, measure the angle of twist. If, however, the specimen’s axis of gravity is shifted or tilted with respect to the fixed axis of rotation during testing, this constrained testing might lead to an error in the measured torsional stiffness due to the parallel axis theorem [11]. The boundary conditions are correctly satisfied only if the torsional test setup fixes the specimens in the torsional degree of freedom while allowing unrestrained movement in the other five degrees of freedom at the site of induced motion. These boundary conditions are very hard to achieve in an experimental setup, which is presumably the reason why, to our knowledge, there is no research group applying pure, unconstrained torsional loading.

Consequently, all methods provide advantages and disadvantages, and it remains unclear which of those is most sensitive to experimental conditions such as alignment of the specimen, directional dependence, or asymmetric mechanical behavior of the specimen. Thus, the aim of this study was to experimentally compare advantages and shortcomings of six different testing methods (*i*.*e*. three-point bending, four-point bending, cantilever bending, axial compression, constrained torsion, and unconstrained torsion) and to find the method with the smallest experimental error.

## Methods

### Test specimens

**Fig 1:**
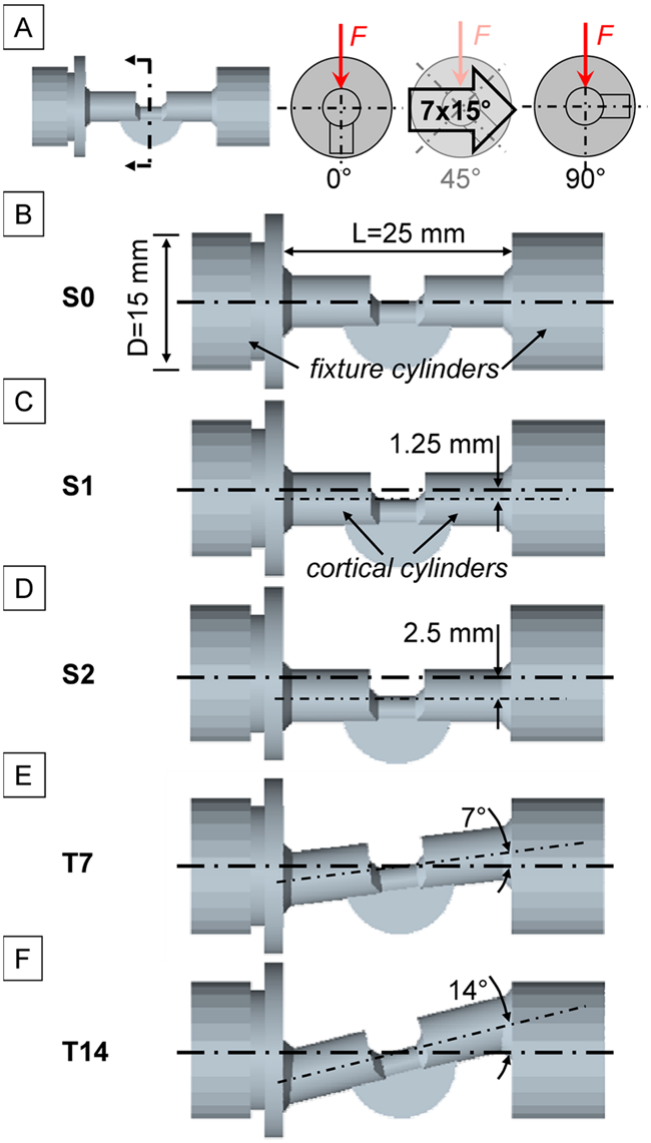
Standardized test specimens representing simplified substitutes for healed rat femurs. A) Variation of the rotational alignment for bending tests in seven increments of 15° between 0°- and 90°-rotational alignment. Model designs showing different relations between the axis of the “fixture cylinders” and the axis of the “cortical cylinders “: B) Design S0 with perfect alignment C) Design S1 with 1.25 mm parallel offset. D) Design S2 with 2.5 mm parallel offset, E) Design T7 with 7° angulation, E) Design T14 with 14° angulation.

To ensure comparability, standardized, simplified substitutes for asymmetrically healed rat femur specimens were (3D-) printed by selective laser sintering (SLS; Beta LAYOUT GmbH, Aarbergen, Germany). The geometry consisted of two “fixture cylinders” with two “cortical cylinders” connected by an asymmetric fracture callus substitute simulated by a semi-circular plate (Fig. 1). The SLS polymer used (PA 2200, EOS GmbH, Krailling, Germany) had a tensile modulus of 1,800 MPa. Since this is an order of magnitude more flexible than real rat cortical bone (tensile modulus of approx. 15,000 MPa according to Wehner *et al*. [31]), the geometry must account for the disparity in the stiffness. Thus, the diameter of the “cortical” section was adapted to reach a bending stiffness (*EI*) of approx. 176805 Nmm^2^ for a length of 25 mm, as was measured for intact rat femurs by Recknagel *et al*. [18]. In a second step, the asymmetric callus substitute was designed to reach a 2-fold difference in bending stiffness between a 0° axial rotational alignment and a 90° axial rotational alignment, representing a direction dependent stiffness behavior as commonly seen for *in vivo* callus formations [29,30,32]. However, this effect was designed to be extreme in order to emphasize the effect of asymmetry. To allow investigation of differences which are dependent on the axial rotational alignment of the specimens, a pattern was added to the fixture cylinders so that the axial rotational angle can be varied in increments of 15 degrees between the 0° and 90° positions (Fig. 1A). Furthermore, five different alignment designs were created to represent differences in embedding of the rat bones (Fig. 1). A perfect alignment (S0) geometry, where the rotational axis of the “cortical cylinders” was congruent with the rotational axis of the “fixture cylinders”, was created as a reference. Furthermore, a moderately shifted (S1) and a maximally shifted (S2) geometry were created with parallel offsets between the cortical and fixture cylinder rotational axes of 1.25 mm and 2.5 mm, respectively. An angulation during embedding of the specimens was simulated by a moderately tilted (T7), and a maximally tilted (T14) geometry, where inclination between cortical and fixture cylinder rotational axes were created with 7°, and 14° angles, respectively. For each testing method, n = 5 separate specimens were used per sample geometry.

### Three-point bending test

**Fig 2:**
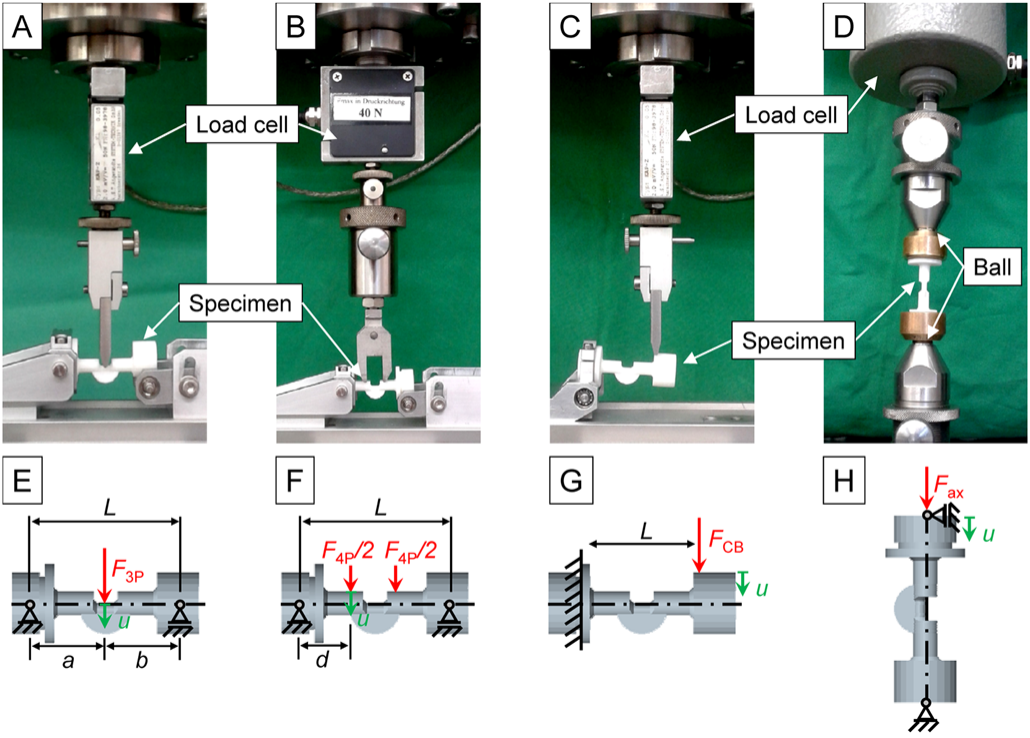
Test setups and boundary conditions for four different experiments: A) and E) three-point bending, B) and F) four-point bending, C) and G) cantilever bending, D) and H) axial compression.

One option for obtaining the flexural rigidity is a non-destructive, three-point bending test (Fig. 2A, E). In this set-up, one fixture cylinder was fixed in a hinge joint, serving as the proximal support for the bending test, while the other cylinder rested on the bending support resulting in a 38 mm effective length (*L*). A quasi-static load was applied in a three-point bending mode with a materials testing machine (1454, Zwick GmbH, Ulm, Germany) using a 50 N load cell (A. S. T. Angewandte System-Technik GmbH, Dresden, Germany). The bending load *F*_3P_ was applied at the center of the specimen geometry and continuously recorded against sample deflection up to a maximum force of 10 N at a crosshead speed of 1 mm/min. After a first settling cycle, flexural rigidity *EI*_3P_ was calculated in the second cycle from the slope *k* of the linear region of the load—deflection curve. The distances between the load vector, the proximal support (*a*), and the distal support (*b*) were kept equal for all specimens (*a = b*). Thereby, the three-point bending stiffness was calculated according to *EI*_3P_ = *kL* ^3^/48 (in Nmm^2^). The stiffness of each specimen was measured in axial rotational alignments of 0° up to 90° in increments of 15°.

### Four-point bending test

Alternatively, a non-destructive, four-point bending test may be used to measure the flexural rigidity (Fig. 2B, F). One fixture cylinder was fixed in a hinge joint, serving as the proximal support for the bending test, whereas the other cylinder rested on the bending support so that a 38 mm effective length (*L*) between the bending supports for the specimen remained. A quasistatic load was applied in a four-point bending mode with a materials testing machine using a 50 N load cell (see above). The bending load *F*_4P_ was applied with two tips of a bracket. The distance (*d*) between each support and the respective neighbor tip was kept constant for all specimens and set to 13 mm. The load was continuously recorded against sample deflection up to a maximum force of 10 N at a crosshead speed of 1 mm/min. Flexural rigidity *EI*_4P_ was calculated from the slope k of the linear region of the load—deflection curve. Thereby, the four-point bending stiffness was calculated according to $EI_{\text{4P}}\text{ = }\frac{kLd^{\text{2}}}{\text{4}}\left(1-\frac{4d}{3L}\right)$ (in Nmm^2^). The stiffness of each specimen was measured in axial rotational alignments of 0° up to 90° in increments of 15°.

### Cantilever bending test

A final, viable option for measuring the flexural rigidity is a non-destructive, cantilever bending test (Fig. 2C, G). One fixture cylinder was fixed in all degrees of freedom, serving as the proximal mounting for the bending test; the other cylinder was left freely suspended. A quasistatic load was applied in a cantilever bending mode with a materials testing machine using a 50 N load cell (see above). The bending load *F*_CB_ was applied with an effective length (*L*) of 28 mm. The load was continuously recorded against sample deflection up to a maximum force of 6 N at a crosshead speed of 3 mm/min. Flexural rigidity *EI*_CB_ was calculated from the slope *k* of the linear region of the load—deflection curve. Thus, the cantilever bending stiffness was calculated according to *EI*_CB_ = *kL* ^3^/3 (in Nmm^2^). The stiffness of each specimen was measured in axial rotational alignments of 0° up to 90° in increments of 15°.

### Axial compression test

Axial compressive stiffness was measured by a non-destructive axial compression test (Fig. 2D, H) using a standard materials testing machine (see above) equipped with a 100 N load cell (Zwick GmbH, Ulm, Germany). Ball bearings located at the top and bottom of the specimen fixture allowed unconstrained rotations. The axial load *F*_ax_ was applied and continuously recorded against sample deflection up to a maximum force of 35 N at a crosshead speed of 0.1 mm/min. After a first settling cycle, the rigidity of axial compression *k*_ax_ was calculated in the second cycle from the slope of the linear region of the load—deflection curve, *k*_ax_ = *F*_ax_/*u* (in N/mm).

### Constrained torsion test

**Fig 3:**
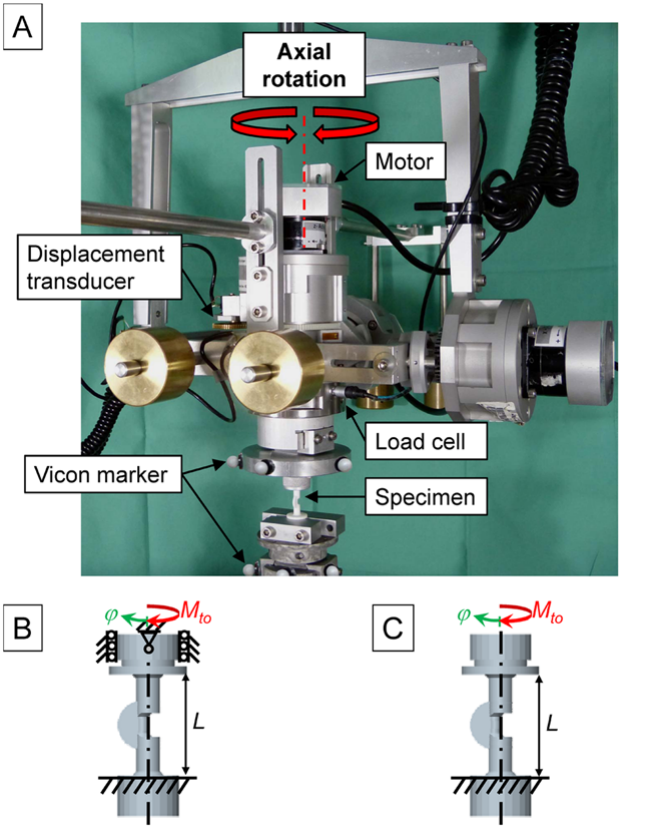
Torsional testing. A) Test set-up for torsional testing in the multi-axial flexibility testing machine. B) boundary conditions for constrained torsional testing (CT) and C) boundary conditions for the unconstrained torsional testing (UT).

Axial torsional stiffness of the bone models was tested in a multi-axis flexibility testing machine [33]. The testing machine consists of a base frame with a 3-axis gimbal. The gimbal is integrated into a three-dimensional slide system enabling unconstrained movements in all six degrees of freedom. Motors integrated into the gimbal allow the application of pure moments in the three principal axes. The three-dimensional bending moments and forces are continuously recorded by a six-component load cell (FT 1500/40, Schunk, Lauffen/Necker, Germany) mounted between the specimen and the gimbal. A set of three retro-reflective markers was fixed to each end of the specimens. Motion of the markers was tracked with 6 motion capturing cameras (Vicon MX 13, Vicon, Oxford, UK). From the motion data, relative motion between the upper and lower fixture was calculated. For the constrained torsion (CT) test, one end of the specimens was rigidly fixed to the base frame of the testing device. The other end was mounted to the gimbal and an axial moment was applied while all secondary axes of motion were fixed. The cups of the specimens were centered to the axis of the motor responsible for axial rotation (Fig. 3A, B). As a starting point, the specimens were positioned in a way that minimized initial, undesired loads. The models were rotationally driven with a rate of 0.5°/s until a rotation angle of 20° was reached over a free torsional length *L* = 25 mm. For evaluation, the torsional stiffness *GI*_CT_ is calculated from the slope *k* of the linear region of the moment—deflection angle curve *GI*_CT_ = *kL* (in Nmm^2^/°).

### Unconstrained torsion test

For the unconstrained torsional test, the same test set-up as for the constrained torsional test was used, as described above with the following modification. During the application of the axial moment, the gimbal was allowed to move freely in all secondary axes of motion (Fig. 3A, C). The resulting torsional stiffness *GI*_UT_ (in Nmm^2^/°) was reported.

### Statistical analyses

To statistically compare the experimental findings with each other, a two-way ANOVA with post-hoc Tukey’s multiple comparison test was performed using GraphPad Prism Software (Version 6.04, GraphPad Software, Inc., La Jolla, CA, USA). Significance level was set to α = 0.05.

## Results

### Three-point bending test

**Fig 4:**
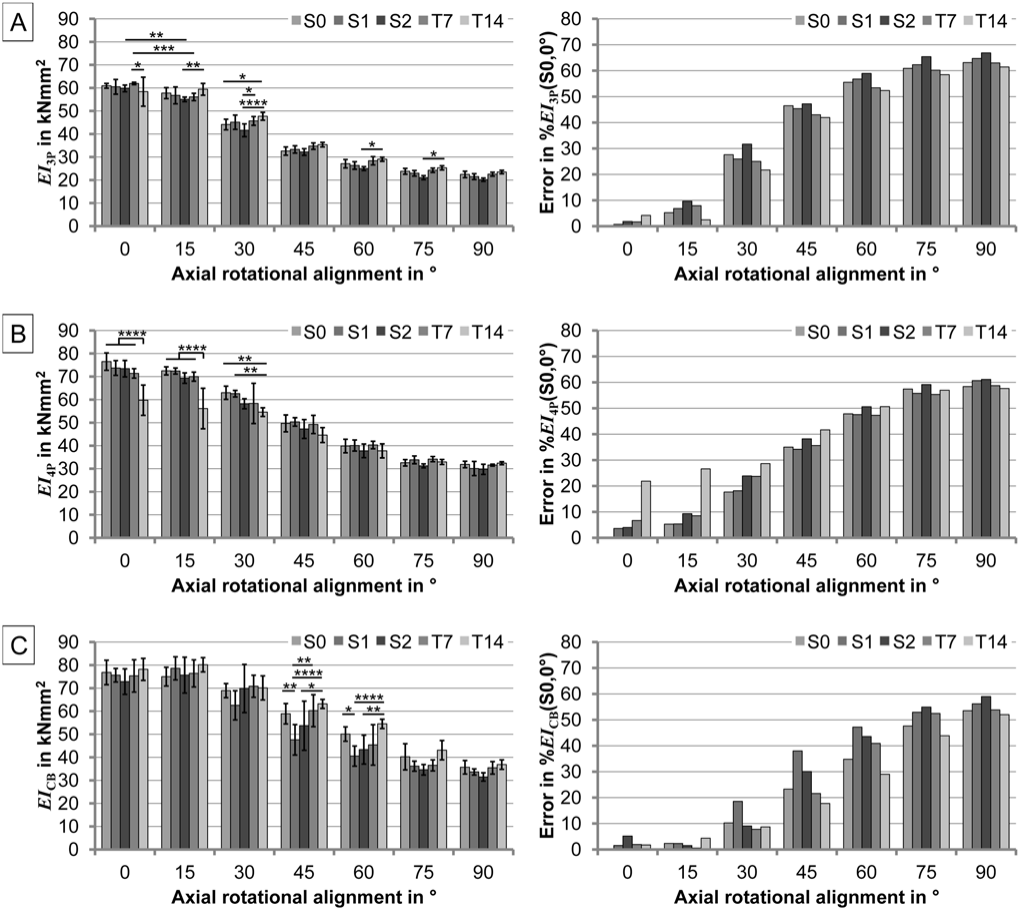
Results for the bending stiffness (mean±SD) (left) and the respective errors of the means (right) in relation to the reference mean value (*i*.*e*. respective mean stiffness of S0 at 0° axial rotational alignment) for three different bending experiments: A) Three-point bending stiffness *EI*_3P_, B) four-point bending stiffness *EI*_4P_, and C) cantilever bending stiffness *EI*_CB_. Results are shown for the five different model designs (S0, S1, S2, T7, and T14) and for the seven different axial rotational angular alignments (0°-90° in 15° increments). *p<0.05, **p<0.01, ***p<0.001, ****p<0.0001.

The results for three-point bending are shown in Fig. 4A, example graphs are included as S1 Fig. For the 0° rotational alignment, the S0 specimens exhibited a bending stiffness *EI*_3P_ of 60928±1038 Nmm^2^ (mean±SD), which decreased to 22459±1394 Nmm^2^, at 90° rotational alignment, giving a relative error of 63.1%. This behavior was found for all five different specimen designs. For each alignment angle, no designs significantly differed in their bending stiffness with the exception of the statistical significances marked in Fig. 4A. For instance, in 0° rotational alignment, only the disparity in bending stiffness between T7 and T14 specimens was noteworthy. Investigating each specimen design for different alignment angles revealed that small deviations from the principal rotational alignments (*e*.*g*. 0° to 15°, or 75° to 90°) showed no significant differences in bending stiffness, except for S2 and T7 specimens between 0° and 15° alignment (Fig. 4A). The maximum error (= 67%) relative to the reference specimen (S0) under 0° rotational alignment was measured for the S2 (maximum parallel offset) specimen under 90° rotational alignment.

### Four-point bending test

The results of four-point bending are shown in Fig. 4B, example graphs are included as S2 Fig. For the 0° rotational alignment, the S0 specimens showed a bending stiffness *EI*_4P_ of 76504±3773 Nmm^2^ (mean±SD), which decreased to 31849±1401 Nmm^2^ at 90° rotational alignment, producing a relative error of 58.4%. This behavior was found for all five different specimen designs. For each alignment angle, no designs significantly varied with the exception of the statistically significant differences marked in Fig. 4B. Thus, in 0° rotational alignment, only the bending stiffness of T14 was significantly different from all other designs. Investigating each specimen design for different alignment angles revealed that small deviations from the principal rotational alignments (*e*.*g*. 0° to 15°, or 75° to 90°) showed no significant differences in the measured bending stiffness for all designs. The maximum error (= 61.1%) relative to the reference specimen (S0) under 0° rotational alignment was measured for the S2 (maximum parallel offset) specimen under 90° rotational alignment.

### Cantilever bending test

The results for cantilever bending are shown in Fig. 4C, example graphs are included as S3 Fig. For the 0° rotational alignment, the S0 specimens revealed a bending stiffness *EI*_CB_ of 76826±5310 Nmm^2^ (mean±SD), which decreased to 35706±2928 Nmm^2^ at 90° rotational alignment, producing a relative error of 53.5%. This behavior was found for all five different specimen designs. For each alignment angle, no designs significantly varied with the exception of the statistically significant differences marked in Fig. 4C. The bending stiffness of some specimens was significantly different for only the 45° and 60° alignment angles. Investigating each specimen design for different alignment angles revealed that small deviations from the principal rotational alignments (*e*.*g*. 0° to 15°, or 75° to 90°) showed no significant differences in the measured bending stiffness for all designs, and an angle up to 30° reveals errors <10% for all designs, except S1. The maximum error (= 59%) relative to the reference specimen (S0) under 0° rotational alignment was measured for the S2 (maximum parallel offset) specimen under 90° rotational alignment.

### Axial compression test

**Fig 5:**
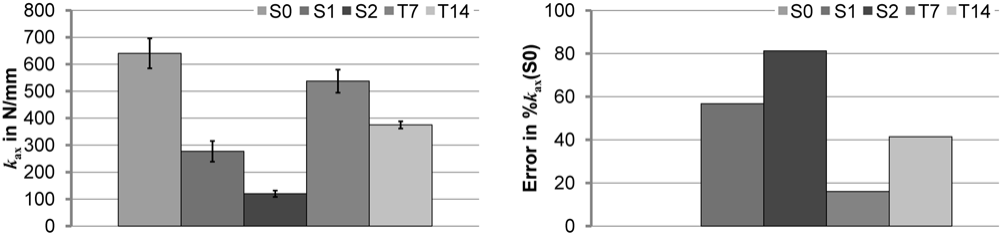
Axial compressive stiffness *k*_ax_ (mean±SD) (left) and the respective errors of the means (right) in relation to the reference mean value (*i*.*e*. respective mean axial stiffness of S0) for the five different model designs S0, S1, S2, T7, and T14).

The results for the axial compressive stiffness of the specimens are shown in Fig. 5, example graphs are included as S4 Fig. The measured values for *k*_ax_ were 641±56 N/mm, 277±39 N/mm, 120±12 N/mm, 537±43 N/mm, and 375±13 N/mm, (mean±SD) for S0, S1, S2, T7, and T14 designs, respectively. The differences between the designs were all significant with p<0.01. The maximum error (= 81.2%) relative to the reference specimen (S0) was measured for the S2 (maximum parallel offset) specimen; tilting created smaller errors (<41.4%).

### Constrained torsion test

**Fig 6:**
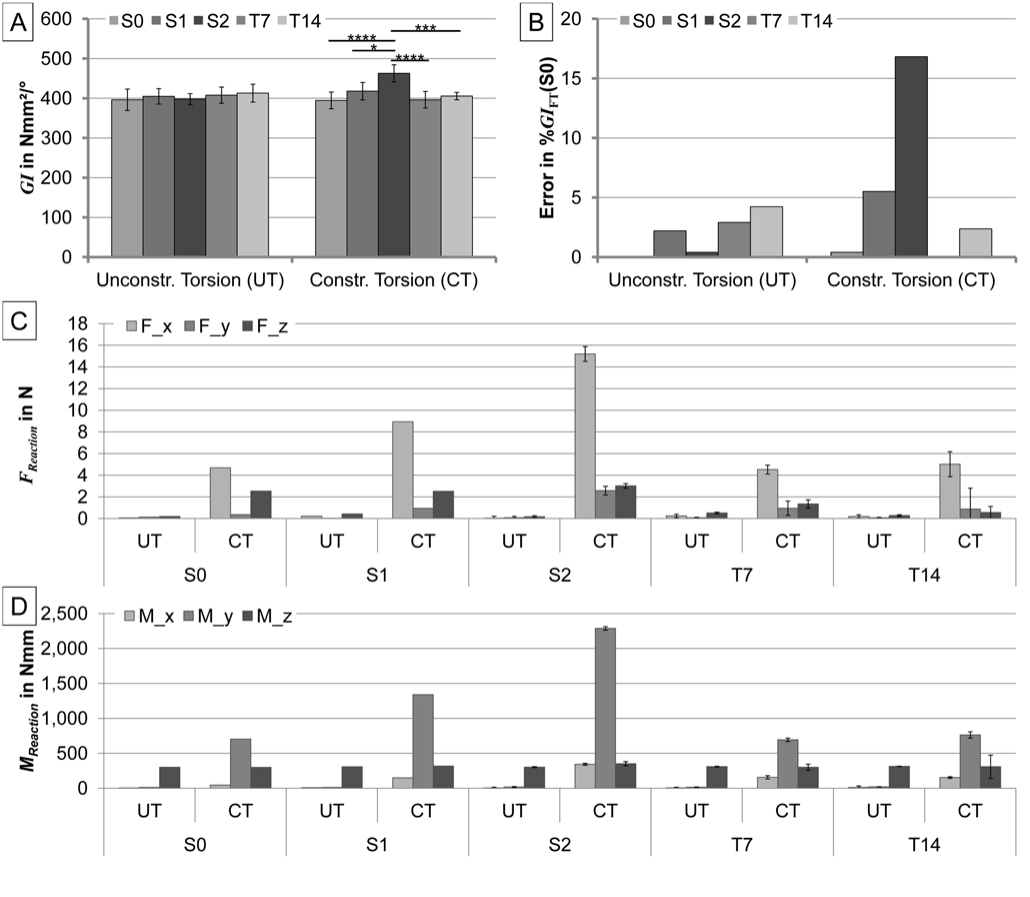
Results of the torsional testing. A) Torsional stiffness *GI* for the five different model designs S0, S1, S2, T7, and T14, under unconstrained (UT-left) and constrained (CT-right) axial torsion. B) The respective errors of the means in relation to the reference mean value (*i*.*e*. stiffness of S0 under unconstrained torsion). C) Reaction forces F at the gimbal in axial direction (z) and the secondary directions (x and y). D) Reaction moments M at the gimbal in axial direction (z) and the secondary directions (x and y). *p<0.05, ***p<0.001, ****p<0.0001.

The torsional stiffnesses *GI*_CT_ measured in constrained torsional testing were 394±21 Nmm^2^/°, 418±22 Nmm^2^/°, 463±22 Nmm^2^/°, 396±21 Nmm^2^/°, and 406±9 Nmm^2^/° (mean±SD), for S0, S1, S2, T7, and T14 specimens, respectively (cf. Fig. 6). The measured torsional stiffness was significantly different solely between S2 and all other specimen designs. Furthermore, large constraining forces and moments (*i*.*e*. the same order of magnitude as in torsional direction) were measured in the secondary directions (Fig. 6C, D). Example graphs are shown in S5 Fig.

### Unconstrained torsion test

The torsional stiffness *GI*_FT_ measured in unconstrained torsional testing was 396±27 Nmm^2^/°, 404±20 Nmm^2^/°, 397±14 Nmm^2^/°, 408±20 Nmm^2^/°, and 412±23 Nmm^2^/° (mean±SD), for S0, S1, S2, T7, and T14 specimens, respectively (cf. Fig. 6). There were no significant differences (p>0.69) in torsional stiffness between the different specimen designs (relative errors compared to S0 specimen were <4.2%). Comparing unconstrained and constrained torsion for each specimen again revealed no significant differences for the torsional stiffness except for the S2 design (p<0.0001). The errors of the means, created by constrained torsion on the different specimen designs resulted in relative errors of 0.4%, 5.5.%, 16.8%, 0%, and 2.4%, for S0, S1, S2, T7, and T14 specimens, respectively, when compared to S0 under unconstrained torsion. The reaction forces and moments were negligible in all secondary directions (Fig. 6C, D). Example graphs are shown in S5 Fig.

## Discussion

### Bending tests

All three bending test setups showed the same principal behavior; for the majority of comparisons, there were no differences in the measured bending stiffness between the different specimen designs for each rotational alignment. This indicates robustness against experimental variations in embedding and fixation of the bone specimens. The rotational alignment plays an important role and shows a high directional dependency for all bending tests, in the worst case leading to maximum relative errors of around 60%. Thus, the direction of the maximum fracture callus expansion relative to the loading direction influences the measured bending stiffness decisively, mechanically defined by the second area moment of inertia. However, the results suggest that small angular deviations from the principal directional alignments had no significant influences on the measured stiffness. Comparing the different setups reveals that variations in the measured bending stiffness were highest for cantilever bending, indicating a lower robustness of the method, however, the errors caused by angular deviations up to 30° were smaller than in the other two bending tests. In four-point bending, large variations occurred only for the T14 design, especially in 0° and 15° alignment (relative errors of >20%), which was due to variations in the positioning of the two tips of the indenter bracket on the strongly tilted surface of the specimen. Absolute values for *EI* were lowest in three-point bending and highest in cantilever bending, which might be due to the differences in the position of the maximum acting bending moment and the effective second moment of the area (*I*) due to the inhomogeneous geometry; in three-point bending the largest bending moment is located at the softer callus material position; in four-point bending the location is at the hard cortical bone, close to the supports; and in cantilever bending, the maximum bending moment is located directly at the rigid fixation. This indicates that for inhomogeneous geometries, the bending stiffness *EI* are not directly comparable without further normalization between different testing methods.

Applying bending tests on real rat bone specimens leads to the problem that the indenter tips might subside into the bone surface and therefore tampers with the indenter displacement measured for bending stiffness calculation. This is of particular importance in three-point bending, when the indenter pushes on the softer callus materials. A solution is to apply several settling cycles before the actual measurement loading cycle, or by the use of external displacement measurement (*e*.*g*. laser) on the unloaded surface of the callus (*i*.*e*. opposite site to the indenter). Four-point bending circumvents this problem to a certain extent, since the indenters are pushing on the stiffer cortical bone. However, due to the small geometries in rats and especially in mice, the small distance between support and indenters leads to incalculable, overlapping shear strain effects dictated by boundary constraints; consequently, this significantly influences the results. For this reason, standards specify the distance between the indenters as a fraction of the effective length *L* (*i*.*e*. 1/3*L* according to ISO 14125, and 1/2*L* according to ASTM D 7264). Furthermore, mechanical testing should preferably induce physiological relevant loading, which in rat femurs is mainly bending and axial loading, whereas shear loads are negligible [34]. Since the largest bending moment in cantilever bending is at the supporting clamp, a standardized testing of the callus stiffness is hard to achieve, because the callus position varies between *ex vivo* specimens. One could solve this problem by embedding the specimens up to the point where the callus begins, however, due to the small geometries of rat and mouse bones, this will induce the previously mentioned boundary constraint errors.

Concluding, three-point bending is easier to apply on small rat and mouse bones under realistic testing conditions. It yields more robust measurements compared to cantilever bending, provided low variation of the callus shape among the tested specimens. To achieve this, highly standardized facture fixation procedures, including plates, intramedullary locking nails or external fixators should be used in small animals studies to provide a defined mechanical environment [1]. The simple intramedullary pin, which remains the most common fixation device due to easy application, cannot provide standardized mechanical conditions and might lead to high variations in the callus geometry [1]. If this occurs, special attention should be paid to the consistent orientation of the specimen’s maximum callus expansion direction relative to the loading direction when performing bending tests.

### Axial compression testing

Axial compression tests are independent of the rotational alignment of the maximum callus expansion direction. Large variability in the axial stiffness was measured for the different specimen designs, indicating strong influences of experimental variations in embedding and fixation of the specimens. The maximum parallel axis offset created a relative errors of more than 80%, most likely due to bending effects within the specimen. However, angulation of the specimens showed smaller effects with maximum relative errors of around 40%. Based on these results, it cannot be recommended to use axial compression for the determination of *ex vivo* fracture callus stiffness of rat (or mouse) long bones.

### Torsional testing

Torsional tests are independent of the rotational alignment of the maximum callus expansion direction. Testing under unconstrained torsional conditions is also independent of the specimen design (relative errors <4.2%), and therefore robust against experimental variations in embedding, but difficult to realize in an experimental setup. The constrained set-up showed some bias, especially for specimens with shifted rotational axes. The larger the parallel offset of the torsional axis in the constrained test, the larger the experimental error became (up to 16.8% for the maximum parallel offset of 2.5 mm). Angulation of the specimens did not significantly influence the test results, particularly if the tilted axis crosses the constrained torsional axis. However, for torsional tests, the specimens need to be embedded on both, proximal and distal ends. This might lead to problems due to the small geometries of rat and especially mouse bones. Although the measurement errors occurring due to the constrained torsional conditions were only significant for larger parallel axis offsets, these may be higher under experimental conditions, when the perfect alignment of the fixture cylinders cannot be realized with small rat or mouse bones due to the embedding of both ends of the long bone.

### General discussion

In the present study, the focus was set on investigating and comparing different established testing methods for determining the *ex vivo* fracture callus stiffness of rat long bones. Therefore, simplified specimens were created, which mimic size, directional behavior, and embedding variations of respective rat bone specimens. Comparing the different testing methods suggests that unconstrained torsional testing is the most robust test method for measuring the callus stiffness, as it is not sensitive to experimental errors due to either embedding variability, or directional alignment of the specimens. Nevertheless, it has the drawback that, in turn, no directional differences of the callus stiffness (*e*.*g*. direction of the maximum callus expansion) can be measured. Since unconstrained torsion is difficult to realize in an experimental setup, to the author’s knowledge, it was not yet been used to measure fracture callus stiffness. Several groups use constrained torsion instead [6,7,8,9,10,11,12,13,14], which is sensitive to embedding uncertainties, especially for shifting of the specimens’ axis of gravity creating a parallel offset to the fixed measurement axis of rotation. Furthermore, bending tests are established for *ex vivo* mechanical testing [15,16,17,18,19,20,21,22,23,24,25]. Bending tests have the potential to deliver robust values of the callus stiffness, independently of embedding errors, provided that the maximum callus expansion direction is consistently oriented relative to the loading direction for all investigated specimens. Whereas small deviations in angular alignment (<15°) showed no alteration of the measurements, larger deviations drastically influenced the measurements and produced relative errors of more than 60%. This indicates a problem for experimental fracture healing studies, in which the direction of the largest callus expansion varies between different animals, even within the same treatment groups. Torsional testing would not show differences in callus stiffness with respect to the callus orientation, whereas bending tests would show large differences, leading to differing conclusions about the mechanical stability of the investigated bone specimens. The directional dependency must be accounted for when performing bending tests and if this differs significantly between the animals, measurement of the stiffness in different rotational alignments should be considered. Otherwise, differences between various *in vivo* test groups may not be detected due to large standard deviations. Compared to torsional loading, bending tests have the advantage of better representing physiological loading [34].

The study presented recognizes several limitations. Firstly, the homogeneous material properties of the specimens are standardized and reproducible. Therefore, this material behaves very differently than real rat bone in which the composition of the callus materials is inhomogeneous and can differ drastically between individuals. On average, the compliance of the material is 10-fold higher than that of rat cortical bone, which universally biases the present results even though the geometry was adapted to achieve the flexural rigidity of rat cortical bone. However, this study attempts to address differences between testing methods, which necessitates the use of standardized, reproducible specimens. Secondly, the geometries of the specimens represent fracture calluses at a late stage of healing, when a large portion of the stiffness has been regained by the callus tissues; early healing stages show more flexible behavior, which might affect the findings. The simplified specimens mimic extreme embedding variations which represent either a parallel axis offset or axis angulation of the specimens in actual experiments; combined offset and angulation was not accounted for. Also the asymmetry of the callus was designed to mimic an extreme scenario. Physiological asymmetries occur more gradually around the rotational axis. Other factors producing variations in real bone specimens, such as bone curvature, fracture location and distance from the supports, specimen size, and the degree and direction of callus asymmetry, were not explicitly investigated in this study, which addresses the principal differences between the different test methods. However, these are important variables to consider when measuring stiffness *ex vivo*, particularly for bending tests. To clarify the points mentioned, the curvature of the bone would likely alter the rotational alignment dependence and the effects of asymmetry by exaggerating or counteracting shifting and tilting within the embedding. The influence of fracture location could compound the issues. Local stiffness at individual locations along the bone will be different due to variations in the moment of inertia of the cross section, the primary geometric parameter influencing stiffness. In the intact bone, more of the total deformation will occur in the locations with the smallest moments of inertia relative to the bending moment at that location. If the fracture occurs through one of these areas, the relative decrease in stiffness may be much lower than the decrease if the fracture and callus formation were to occur through a location with a relatively large cross section. The areas with large cross sections previously contributed much of the overall stiffness. Furthermore, shifting the location of the fracture relative to the supports will lead to a difference in the bending moment at the location of the callus and a higher influence of the boundary conditions thereby further influencing the measurements. Along the same reasoning, since stiffness is an extrinsic property of a structure depending on both the material and the geometry, variations in the size of either the effective length or cross section between physiological specimens will alter the measured stiffness. Likewise, as it has been shown that callus asymmetry substantially influences the results in bending tests, it is logical that changes in the asymmetry between specimens in both magnitude and direction will further complicate analysis. Thirdly, the specimens were created with perfectly aligned fixture cylinders on both sides of the specimen, which is hard to realize when embedding the *ex vivo* bones in distal and proximal fixation cups. Lastly, as in other experimental studies [35,36], the applied methods actually measured the whole-bone stiffness of the specimens and declare it as the callus stiffness. Further investigation of the direct relation between whole-bone and callus stiffness is needed.

## Conclusion

This study investigated the differences between and deficiencies of several methods for biomechanical assessment of fracture callus stiffness in small animal models. Each method has its shortcomings and advantages, the most robust method was found to be unconstrained torsional testing, which however is experimentally difficult to realize. Still, constrained torsion produced robust results with only small relative errors due to embedding uncertainties, except in cases of extreme parallel offset. Axial compressive testing was very sensitive to embedding uncertainties leading to the largest measurement errors of >80%. Bending tests are generally more robust against embedding uncertainties and represent more physiological loading. They show large dependency on the direction of the maximum fracture callus expansion relative to loading direction, however small angular deviations lead to negligible experimental errors. Subsidence of bending indenters can be addressed by external displacement measurement or by applying several settling cycles. Due to the small geometries in rat and mouse bone, four-point bending is difficult to perform and may create boundary constraint errors. Three-point bending is easier to apply on small rat and mouse bones under realistic testing conditions and yields robust measurements, provided low variation of the callus shape among the tested specimens.

## Supporting Information

S1 Fig Example graphs for three-point bending. Each specimen (S0, S1, S2, T7, T14) was tested in 7 different angular alignments (0°-90°).
(PDF)

S2 Fig Example graphs for four-point bending. Each specimen (S0, S1, S2, T7, T14) was tested in 7 different angular alignments (0°-90°).
(PDF)

S3 Fig Example graphs for cantilever bending. Each specimen (S0, S1, S2, T7, T14) was tested in 7 different angular alignments (0°-90°).
(PDF)

S4 Fig Example graphs for axial compression. (PDF)

S5 Fig Example graphs for torsional testing under constrained and unconstrained boundary conditions. (PDF)
