# Supplementary figures and images for: Comparison between Different Methods for Biomechanical Assessment of Ex Vivo Fracture Callus Stiffness in Small Animal Bone Healing Studies
Source: PLoS One. 2015 Mar 17;10(3):e0119603. doi: 10.1371/journal.pone.0119603 (PMC4363594; doi:10.1371/journal.pone.0119603)

# Three-Point Bending

**S0**

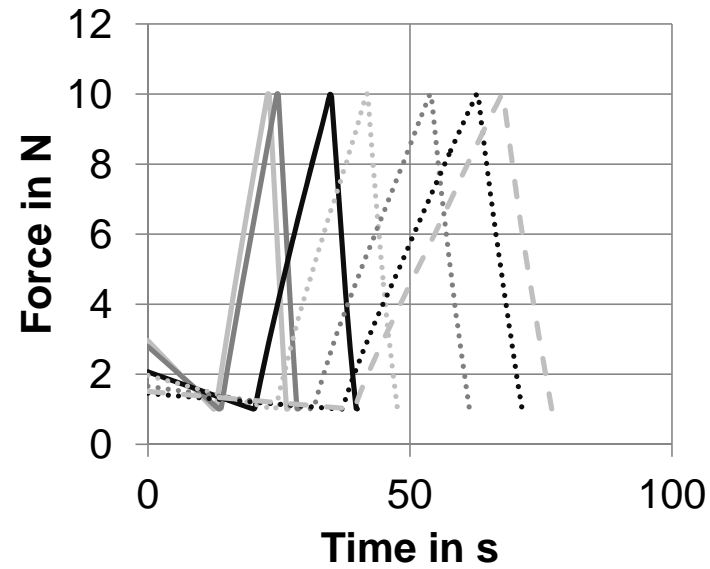

**S1**

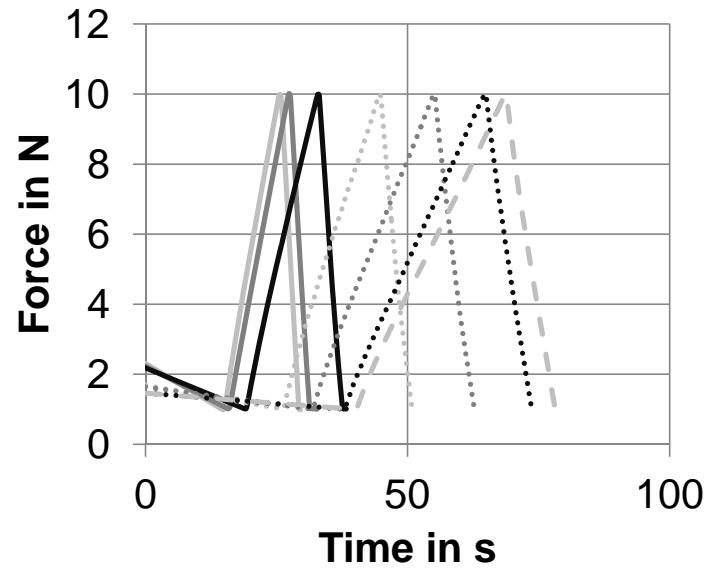

**S2**

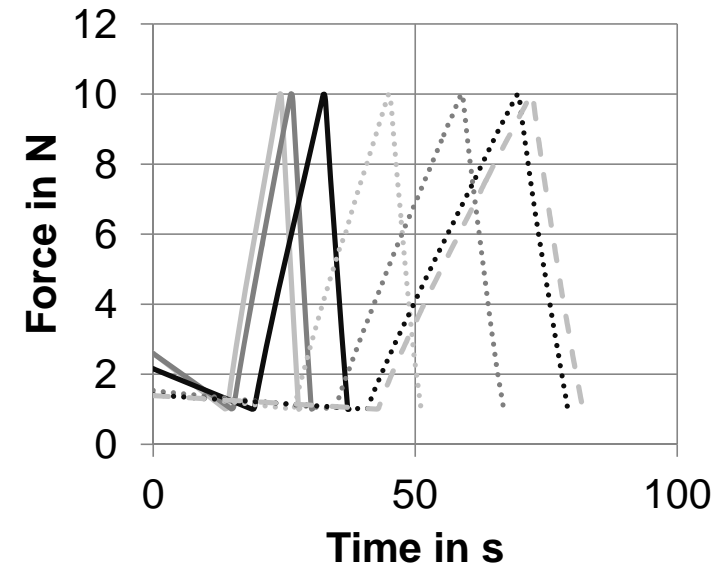

**T7**

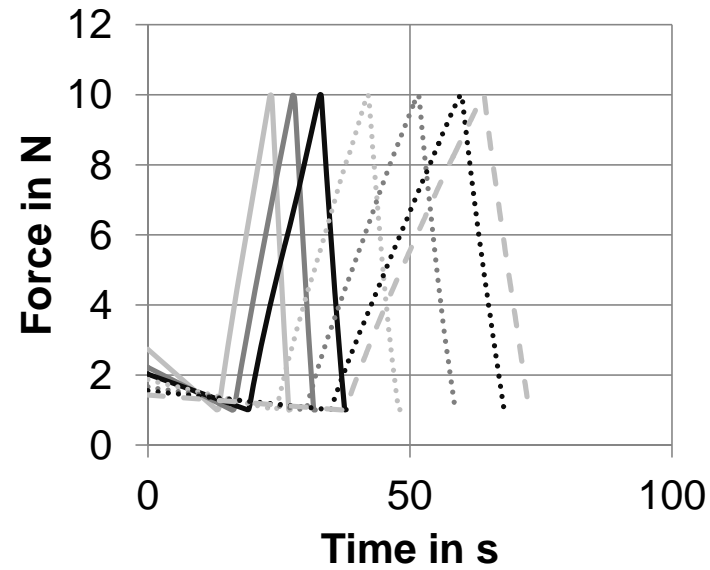

**T14**

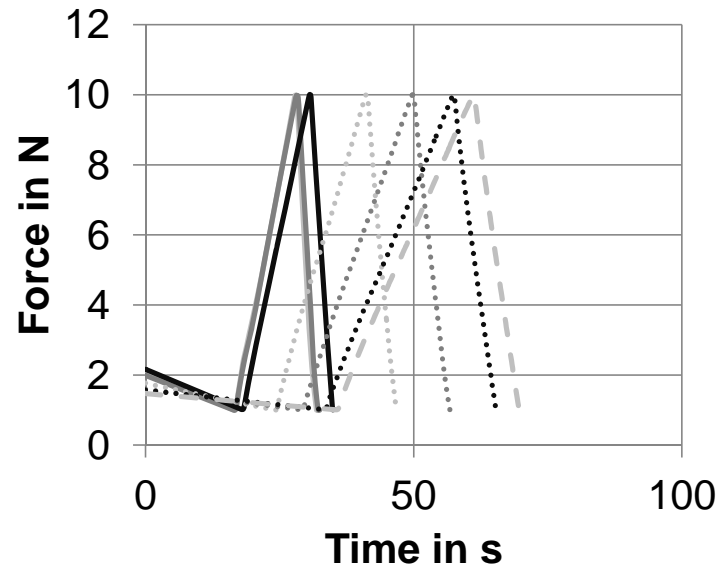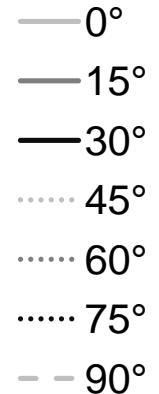

Supplement: S1 Fig — Each specimen (S0, S1, S2, T7, T14) was tested in 7 different angular alignments (0°-90°). (PDF) [file pone.0119603.s001.pdf]

# Four-Point Bending

**S0**

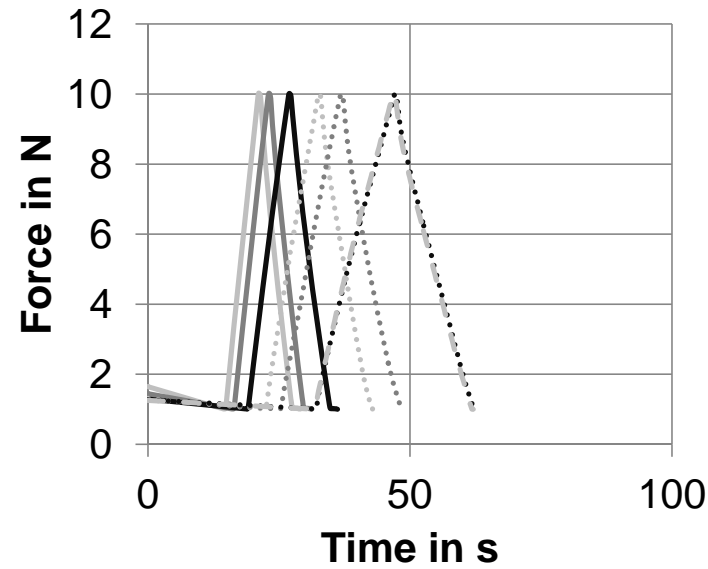

**S1**

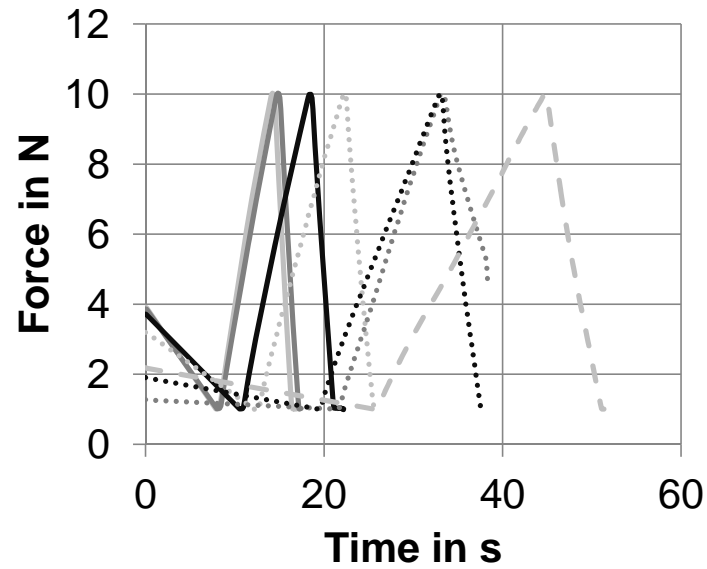

**S2**

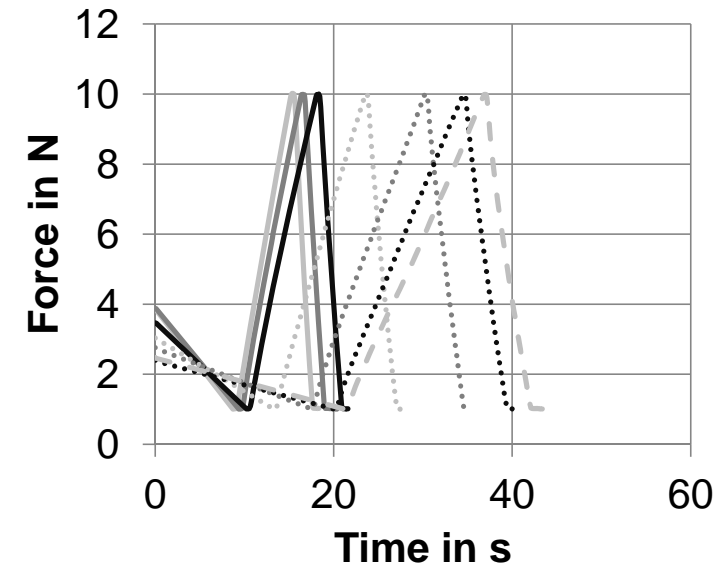

**T7**

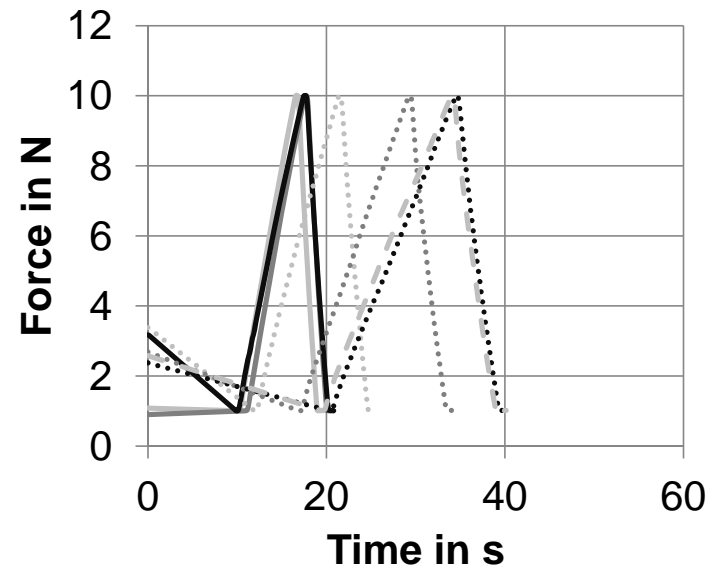

**T14**

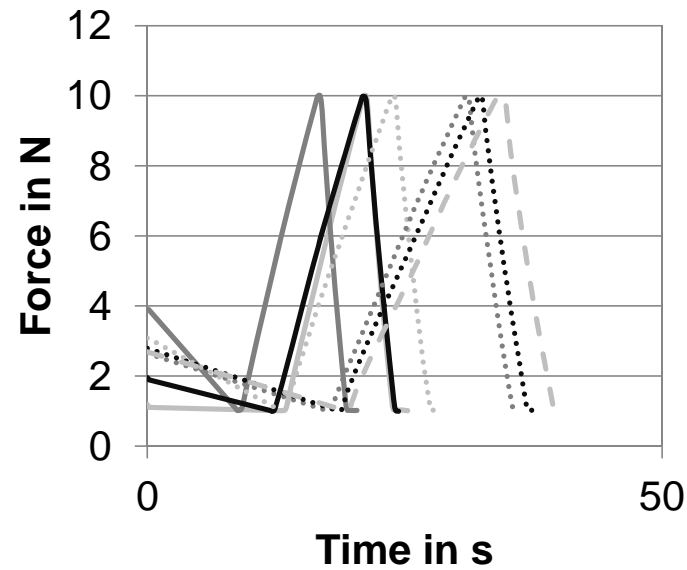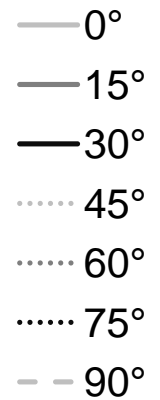

Supplement: S2 Fig — Each specimen (S0, S1, S2, T7, T14) was tested in 7 different angular alignments (0°-90°). (PDF) [file pone.0119603.s002.pdf]

# Cantilever Bending

**S0**

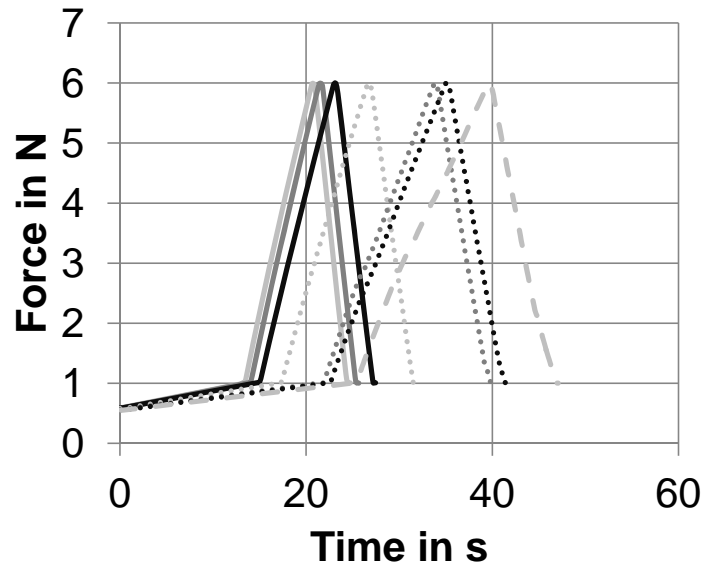

**S1**

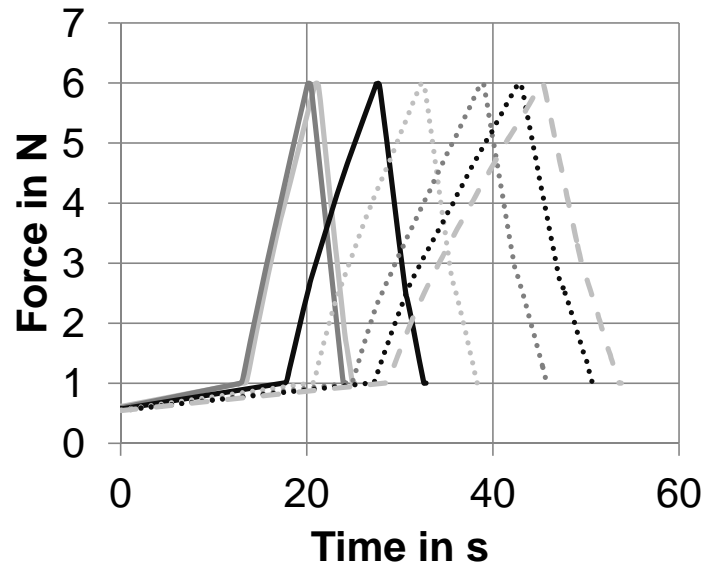

**S2**

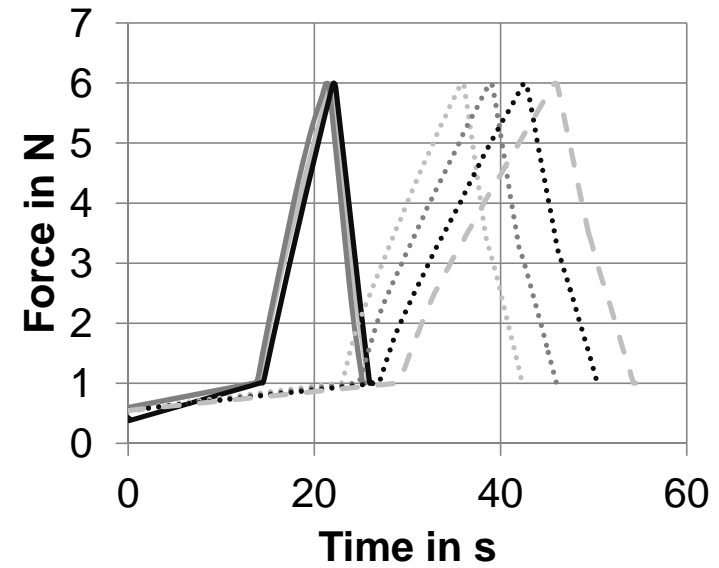

**T7**

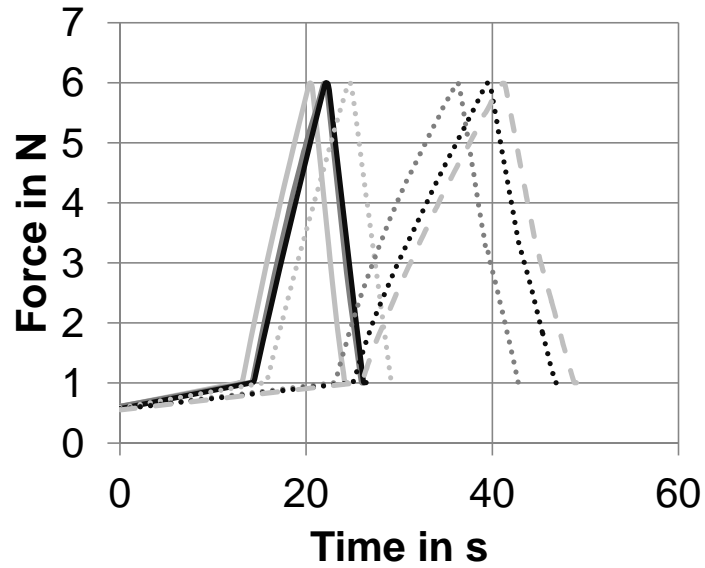

**T14**

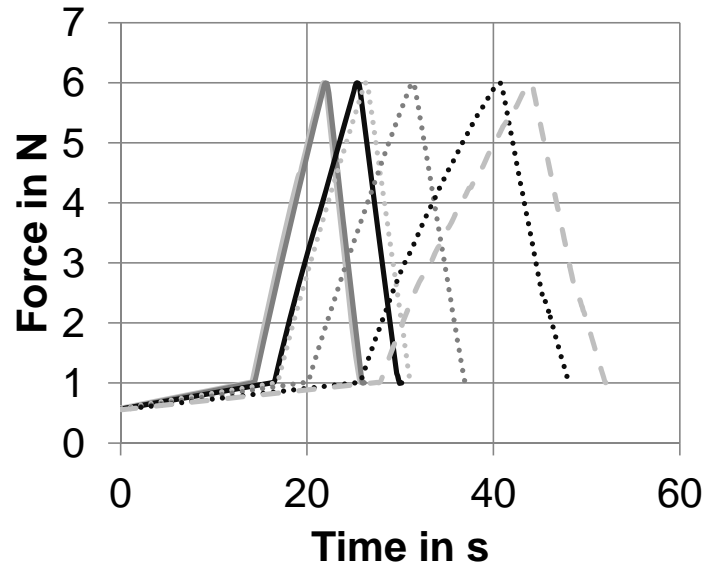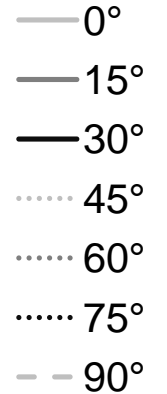

Supplement: S3 Fig — Each specimen (S0, S1, S2, T7, T14) was tested in 7 different angular alignments (0°-90°). (PDF) [file pone.0119603.s003.pdf]

# Axial Compression

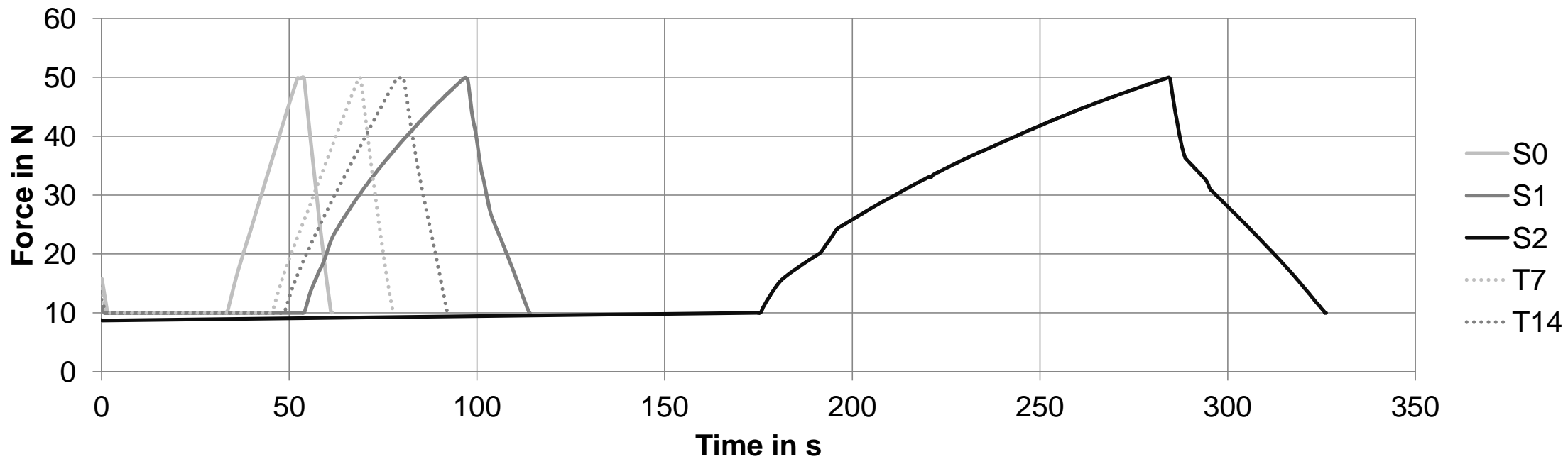

Supplement: S4 Fig — (PDF) [file pone.0119603.s004.pdf]

# Torsional Rotation

**S0**

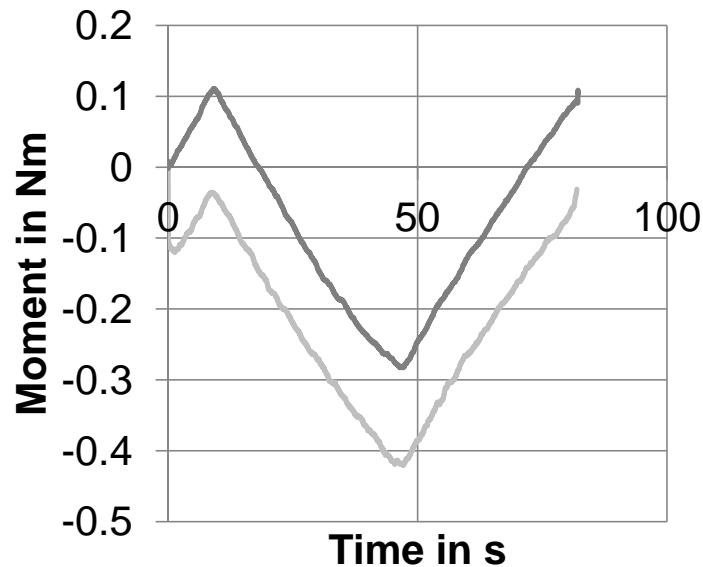

**S1**

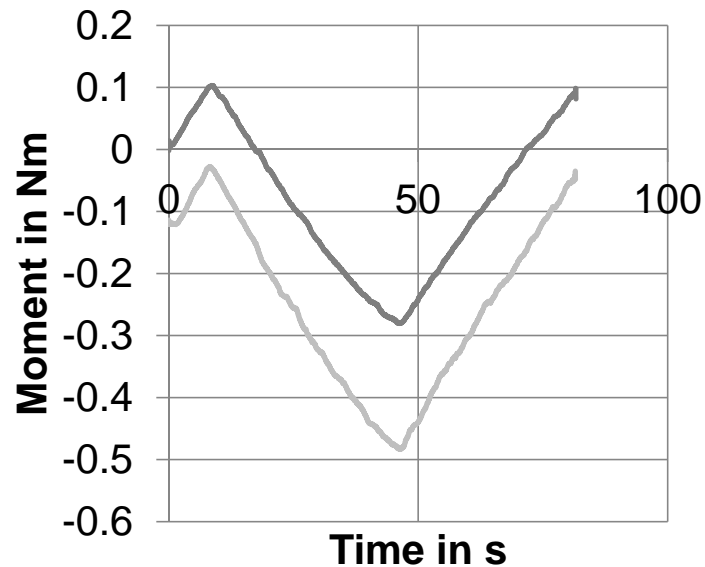

**S2**

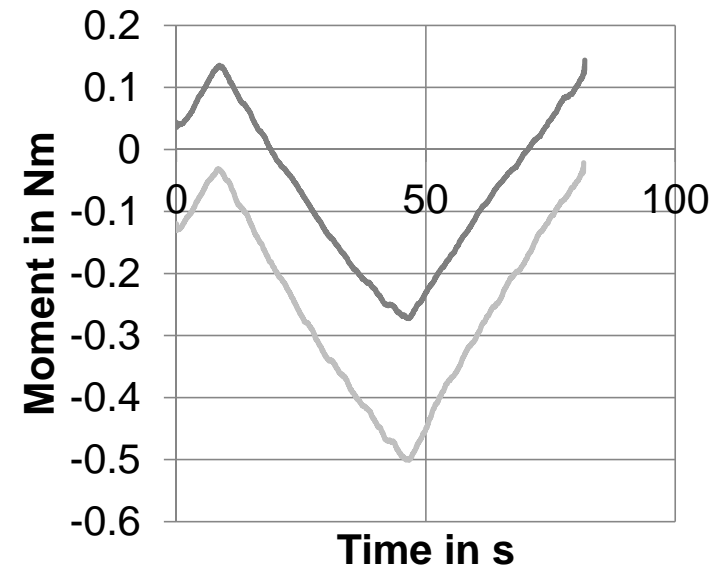

**T7**

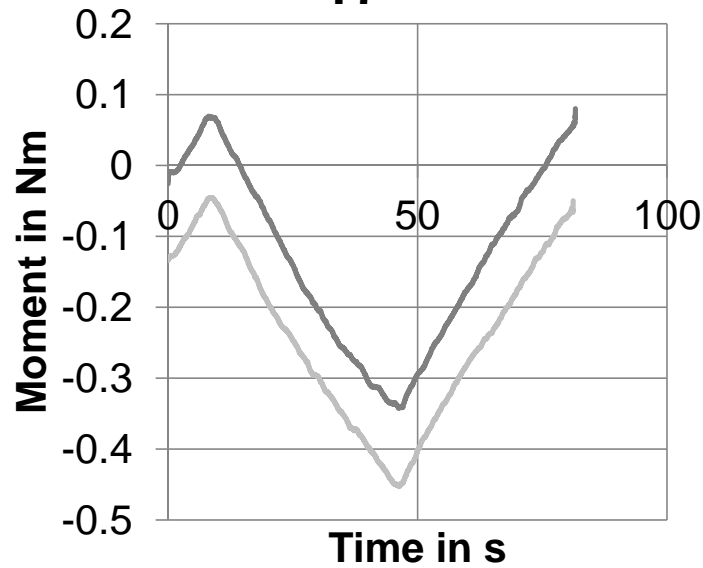

**T14**

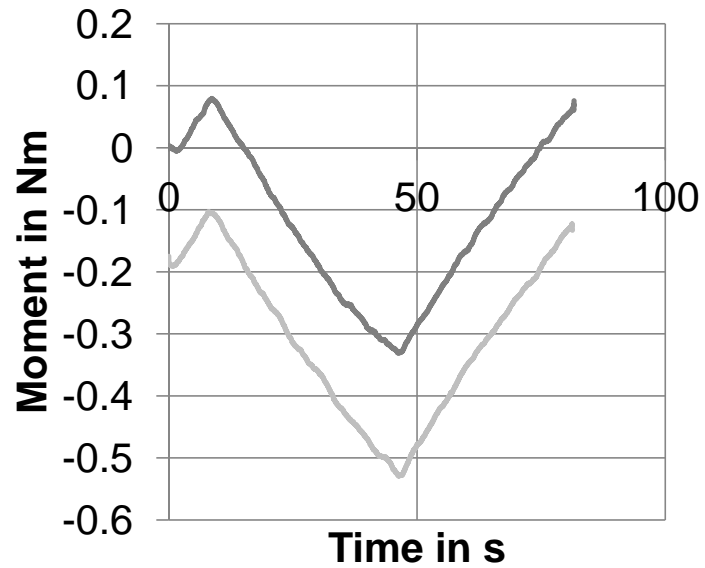

— Constrained  
— Unconstrained

Supplement: S5 Fig — (PDF) [file pone.0119603.s005.pdf]
